# From aggregation to interpretation: how assessors judge complex data in a competency-based portfolio

**DOI:** 10.1007/s10459-017-9793-y

**Published:** 2017-10-14

**Authors:** Andrea Oudkerk Pool, Marjan J. B. Govaerts, Debbie A. D. C. Jaarsma, Erik W. Driessen

**Affiliations:** 1 Department of Educational Development and Research, Maastricht University, Universiteitssingel 60, 6229 ER Maastricht, The Netherlands; 2 Center for Education Development and Research in Health Professions (CEDAR), Faculty of Medical Sciences, University Medical Center Groningen, University of Groningen, Groningen, The Netherlands

**Keywords:** Assessment, Competency-based medical education, Information processing, Portfolio, Rater cognition, Think-aloud method, Undergraduate medical education

## Abstract

While portfolios are increasingly used to assess competence, the validity of such portfolio-based assessments has hitherto remained unconfirmed. The purpose of the present research is therefore to further our understanding of how assessors form judgments when interpreting the complex data included in a competency-based portfolio. Eighteen assessors appraised one of three competency-based mock portfolios while thinking aloud, before taking part in semi-structured interviews. A thematic analysis of the think-aloud protocols and interviews revealed that assessors reached judgments through a 3-phase cyclical cognitive process of acquiring, organizing, and integrating evidence. Upon conclusion of the first cycle, assessors reviewed the remaining portfolio evidence to look for confirming or disconfirming evidence. Assessors were inclined to stick to their initial judgments even when confronted with seemingly disconfirming evidence. Although assessors reached similar final (pass–fail) judgments of students’ professional competence, they differed in their information-processing approaches and the reasoning behind their judgments. Differences sprung from assessors’ divergent assessment beliefs, performance theories, and inferences about the student. Assessment beliefs refer to assessors’ opinions about what kind of evidence gives the most valuable and trustworthy information about the student’s competence, whereas assessors’ performance theories concern their conceptualizations of what constitutes professional competence and competent performance. Even when using the same pieces of information, assessors furthermore differed with respect to inferences about the student as a person as well as a (future) professional. Our findings support the notion that assessors’ reasoning in judgment and decision-making varies and is guided by their mental models of performance assessment, potentially impacting feedback and the credibility of decisions. Our findings also lend further credence to the assertion that portfolios should be judged by multiple assessors who should, moreover, thoroughly substantiate their judgments. Finally, it is suggested that portfolios be designed in such a way that they facilitate the selection of and navigation through the portfolio evidence.

## Introduction

With the rise of competency-based assessment, portfolios are increasingly seen as the linchpin of assessment systems. Although their format and content may differ, generally they all contain reporting on work done, feedback received from peers and faculty, progress made, and goals and plans on how to further improve competence (Driessen et al. 2007).

Worldwide, multiple medical schools have implemented competency-based assessment systems in which the portfolio is key to the assessment of students’ achievements (Dannefer and Henson 2007; Davis et al. 2001; Driessen 2016; Smith et al. 2003). In these portfolio-based assessment systems, decisions regarding the students’ level of competence typically rely on expert judgment. It is assumed that expert judges are able to select, interpret, and integrate relevant evidence in the portfolio, and consequently make a valid decision about a student’s competence.

Evidence to support assumptions about assessors’ decision-making is rather limited. Although prior research has addressed the question of how assessors develop judgments, the latter concerned judgments based on direct observations (Kogan et al. 2011) or single assessments, forgoing the opportunity to investigate how holistic judgments are formed on the basis of complex data collected in the student’s portfolio.

Moreover, recent studies on in-training evaluations revealed a discrepancy between what faculty see as important qualities in a future clinician, and the roles defined within competency-based assessment (Ginsburg et al. 2011; Renting et al. 2016; Rosenbluth et al. 2014). When asked to assess students’ level of clinical competence, faculty assigned varying degrees of importance to certain aspects depending on the resident: shortcomings of exceptional students could be discounted while strong attributes of weaker students were overlooked (Ginsburg et al. 2010). Besides, some constructs that were of importance in the considerations of assessors were not even competencies at all. For example, assessors attached great importance to how the student affected the supervisor (coined ‘impact on staff’). Hence, there seems to be a mismatch between the content of competency frameworks and the aspects that clinicians consider important.

Previous research on portfolio assessment in, for instance, teacher education has furthermore demonstrated that even assessors who hold a shared vision of effective teaching and who cite much the same evidence can, nonetheless, develop significantly different ‘stories’ or interpretive summaries of performance (Schutz and Moss 2004).

These aforementioned research findings and increasing importance of portfolio-based assessments (Driessen 2016) call for an exploration of assessors’ information processing when interpreting and valuing complex competence data in a portfolio. The purpose of the present study is therefore to explore assessors’ judgment and decision-making processes when interpreting evidence from various sources and multiple performance data in a competency-based portfolio. Findings may improve portfolio-based assessment practices.

## Methodology

### Setting

The research was set in the Master’s in Medicine (MiM) program of Maastricht University, the Netherlands. The MiM curriculum spans a 3-year period following the bachelor’s in Medicine. It consists of clerkships, a research project, and electives. The curriculum has been designed according to the principles of competency-based education and assessment, using the CanMEDS framework as overarching assessment framework (Frank and Danoff 2007). Competency-based assessment is supported by a web-based portfolio system in which students collect and reflect on evidence of their learning and development in each of the competency domains (Moonen-van Loon et al. 2013). Every student is assigned a mentor who monitors the student’s competency development by guiding the student in his or her self-assessments and reflections, and in setting learning goals. Mentor and student meet three to four times per year, during which the mentor discusses the competency development and portfolio with the student. At a specific point in time the mentor must also assess the student’s competency development and send an advisory judgment to the portfolio assessment committee which makes a formal pass–fail decision.

### Participants

We purposefully selected 18 mentor-assessors using maximum variation sampling (Patton 1990). To maximize variation in assessors’ medical backgrounds, we selected assessors from different medical specialties (Family Medicine and surgical as well as non-surgical specialties). All these participants had experience of the portfolio system and had received training which included instructions on how to use and assess the portfolio. Participants did not receive any additional training for this study.

### Student portfolio

For the purpose of this study, the research team (consisting of a psychologist, two educationalists and a veterinarian) developed three mock portfolios representing three different student profiles, each reflecting varying levels of competency achievement. In our student profiles we chose to make a distinction between de medical expert competency and other competencies because previous research has shown that assessors have difficulty assessing the non-medical expert competencies (Whitehead et al. 2015). Furthermore we included a student profile containing predominantly positive narratives and qualifications in all competency domains because this resembles the profile of a large portion of students. By manipulating the competency data, we created the following portfolios and ensuing student profiles: portfolio (A) predominantly positive feedback in the medical expert domain, but both critical and positive feedback in the domains of manager and communicator; portfolio (B) both critical and positive feedback in the domain of medical expert, but predominantly positive feedback on the other competencies; and portfolio (C) predominantly positive feedback in all domains.

**Fig. 1:**
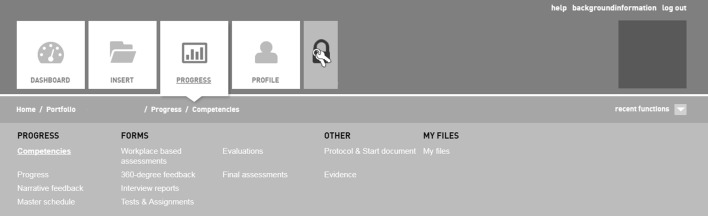
Print screen of the content and organization of the digital portfolio environment used in this study. The portfolio is organized according to the CanMEDS competencies

The portfolios contained evidence on a student’s competencies collected during a single 18-week clinical rotation, including student’s self-assessments, workplace-based assessments (mini-CEXs, DOPSs, field notes, multi-source feedback), progress test results, and a curriculum vitae. Each portfolio comprised narrative feedback, competency ratings and qualifications (i.e., insufficient, sufficient, and good) as well as test results pertaining to each of the individual CanMEDS competencies. Figure 1 provides a print screen of the online portfolio environment used for this study.

Two recent medical graduates provided feedback on the first portfolio drafts. We also invited three assessors to take part in a dry run prior to data collection, to ensure that the information and instructions were clear, and the portfolio versions were authentic, and fit for the study purpose. On the basis of these dry runs and student feedback, we made some adaptations to the portfolios and constructed the final portfolios.

### Ethical approval

We obtained ethical approval from the Ethical Review Board of the Netherlands Association for Medical Education (ERB-NVMO file number 474).

### Data collection and analysis

Data were collected between October, 2015 and January, 2016.

We invited 24 mentor–assessors via e-mail to participate and obtained their consent prior to participation. Eighteen assessors responded to our invitation. Each assessor was presented one of the three portfolio versions, each portfolio version was therefore assessed by six assessors. Assessors were instructed to carefully read it and provide a holistic judgment of the student’s overall professional competence by rating it as ‘insufficient,’ ‘sufficient,’ or ‘good.’ Although assessors were allowed to comment on individual competencies, they did not need to rate each competency domain separately. We did not instruct assessors on how to evaluate the portfolio; they were free to read or skip any portfolio evidence as they deemed appropriate.

To capture assessors’ cognitive processing during portfolio evaluation, we employed the think-aloud method (Van Someren et al. 1994), which means that we instructed assessors to verbalize all their thoughts, ideas, and decisions while reading and evaluating the portfolio. If they fell silent for more than a few seconds, we reminded them to keep verbalizing their thoughts.

When the assessors indicated that they had finished reviewing the portfolio, we conducted a short, semi-structured interview. The first question was to provide a holistic judgment of the student’s competence based on what assessors had found in the student’s portfolio. Additional questions were aimed at encouraging the assessor to reflect on the portfolio assessment process (e.g., did the portfolio provide sufficient information to make a judgment? Did you notice anything unusual about the portfolio?).

All sessions were audiotaped and transcribed verbatim.

In the next phase, we performed a thematic analysis of the think-aloud and interview transcripts (Fereday and Muir-Cochrane 2006). The first author (A.O.P) read the first three transcripts and developed an initial coding manual, on the basis of which a research assistant (C.N.) then coded the same transcripts again. Subsequently, A.O.P. and C.N. compared their results and further refined the coding scheme. After the first seven transcripts were coded, research team members A.O.P., M.G., E.D., and D.J. identified initial patterns by clustering individual codes into broader themes. A.O.P. and C.N. applied the new coding manual to interviews 8–15. The coding manual that ensued was consequently discussed with the entire research team (A.O.P., M.G., E.D., D.J., and C.N.). Theoretical saturation (Bowen 2008) was reached after transcript 12. To make sure that no relevant information had been missed, A.O.P. and C.N. reread all transcripts. Finally, an analysis and coding of transcripts 16, 17, and 18 confirmed the final themes, from where we moved to making a tentative interpretation of how assessors judge a student’s professional competence based on aggregated data collated in a competency-based portfolio.

## Results

We observed that assessors went through a similar process of selecting and interpreting portfolio evidence, while we also noted variations in assessors’ approaches to reaching a judgment. In the next sections we will first describe this shared process and then assessors’ divergent approaches, followed by three explanations for this variance as inferred from the data.

### Assessors’ information processing: a 3-phase cyclical process

In processing information, all assessors followed a similar cyclical pattern of acquiring, organizing, and integrating information, respectively. During the first phase, assessors selected the information they considered the most important and credible pieces of evidence upon which to base their judgment. After reviewing this information, they defined if and how it contributed to an informed judgment about aspects of student’s competence. Assessors subsequently weighed the various sources of evidence and decided on a (preliminary) judgment of the student’s competence.

Upon conclusion of the first round, assessors reviewed the remaining portfolio evidence to look for additional confirming or disconfirming data thereby repeating the information acquisition phase which, in turn, influenced the organization and integration of information. This iterative process was repeated every time the assessor reviewed new portfolio evidence, until assessors felt they had obtained enough information to make a judgment about the student’s competence. By comparing different pieces of evidence from multiple sources, assessors gradually came to recognize patterns in the student’s competence.

A salient finding, moreover, was that assessors were inclined to stick to their initial judgments even when confronted with seemingly disconfirming evidence: Although their final judgments were, indeed, more elaborate and detailed compared to their preliminary judgments, they were not substantially different from their initial judgments. Differences between student profiles did not seem to affect the judgment process or assessors’ overall judgment of the student’s competence: Most assessors rated the students’ competence as sufficient.

### Assessors’ idiosyncratic approaches to the student evaluation

Analysis of the think-aloud protocols revealed that, from the onset of the judgment process, assessors relied on different kinds of portfolio evidence to inform their judgment. Likewise, the amount of portfolio evidence assessors took into account to arrive at decisions about student competence exhibited between-assessor differences: While some assessors read the entire portfolio before providing their final judgment, others mainly relied on either the student’s self-evaluation or workplace-based assessment data to inform their judgment, largely ignoring additional portfolio evidence.

These divergent approaches were rooted in assessors’ varying assessment beliefs, performance theories (i.e., conceptualizations of what constitutes performance effectiveness and professional competence), and inferences. As a result, assessors’ reasoning behind their judgments and judgments of individual competencies were strongly governed by their unique personal profiles and differed accordingly. The following paragraphs will discuss each of these three inter-assessor differences in more detail.

### Differences in what assessors believed to be credible portfolio evidence

First it should be noted that assessors mainly relied on narrative feedback to inform their judgment, because this provided meaningful and detailed information about the student’s development, strengths and weaknesses, as well as specific suggestions for improvement as provided by others. Grades and qualifications were merely used to confirm impressions based on narratives. For example, if assessors suspected insufficient competence within the medical expert competence domain, they would purposefully select those workplace-based assessments for which the student had received an insufficient score because this would probably provide more insight into the reasons for their underperformance.

Despite this commonality, assessors had varying assessment beliefs about what kind of narrative evidence gave the most valuable and credible information about the student’s competence.

Assessors, for instance, chose different pieces of narrative evidence to start their evaluation: some selected narrative comments on workplace-based assessments, believing that these would generate the most authentic evidence of students’ abilities; Other, however, started reading the student’s self-evaluations and reflective writings as they assumed these would contain reference to salient feedback comments and assessment forms which they consequently read to check if the student’s claims were justifiable.

The source also appeared to matter in deciding on the credibility of evidence: Some assessors mainly relied on feedback from physicians because they perceived them as content experts most likely to provide accurate and meaningful feedback on student competence; Others, in contrast, preferred feedback from fellow students and nurses who, they presumed, had worked more closely with students and therefore had more opportunities to directly observe them.

Between-assessor differences in source preferences also stemmed from assessors’ frames of reference and the presumed impact of student-supervisor relationships. During the interviews, for instance, multiple assessors expressed their belief that student’s progress could be established more reliably when the student had received feedback from the same person at different points in time, especially since they found it hard to interpret fragmented and divergent feedback:

At least in my department we try as much as possible to match the interns with one staff member. And also strictly have this person assess presentations and CATs [Critical Appraisals of a Topic] and those sort of things. What you clearly see then, is a pattern and that actually became much less evident from those mini-CEX assessments.

(Assessor 4)

At the same time, other assessors did value the input from multiple assessors, which they estimated to be more reliable and more informative compared to single-person feedback. In explaining their preferences, several assessors invoked perceptions of selection bias (i.e., students purposively selecting more lenient assessors to provide feedback) and feedback providers’ reluctance to write down negative comments so as to avoid conflicts:

As a student, in our department at least, you can be quite selective in who you ask […] So you yourself can choose to team up with your buddies and they give you positive feedback. But in the case of such a 360-degree assessment. That is very comprehensive. And anonymous. And that is, that does give you, I think, the most truthful answers.

(Assessor 11)

Finally, the assessment data in the portfolio also induced different impressions about the quality of supervision. When a supervisor, for instance, failed to provide detailed written feedback, several assessors assumed that the supervisor had probably written down the essential comments and had provided more elaborate feedback verbally. Others, however, believed that the student had gone unobserved and therefore questioned the credibility of the supervisors’ assessments.

### Differences in interpretations of what constitutes ‘competence’

We also observed inconsistencies between assessors’ conceptualizations of ‘competence’. As a result of these variable interpretations of what constitutes competence, assessors thought differently about what they needed to know about the student to be able to form a judgment. Interestingly, these so-called ‘performance theories’ tended to deviate from the formal assessment criteria. In the following we will outline the performance theories that prevailed.

One group of assessors defined students’ competence in terms of the extent to which they actively engaged in their own learning process and effectively used feedback for competence improvement. More specifically, they considered active engagement in learning and assessment a key quality of a good student. Hence, to establish growth, they often read the portfolio evidence in chronological order to check if aspects that did not go well in the beginning of the clinical rotation had improved over time. In the same fashion, they screened the workplace-based assessments and feedback to verify whether the student had followed-up on all the aspects that needed to improve. In their perusal, they also included the student’s self-assessment and learning goals as they felt that it was vital to know if students did follow up on learning goals and appointments. According to this group of assessors, the student’s competence did not necessarily have to be up to standard as long as there was enough evidence that the student had sufficiently improved over time and actively tried to improve:

What I expect the student to do is to make a strengths/weaknesses analysis of the competencies before the internship, a plan of action, like “how am I going to pay attention to that analysis, those strong points?”. In the interim or final assessment an answer to those questions, “Did I accomplish that? Did I live up to that? And why not?” And “What is my assessment of myself when I look at myself, am I satisfied and have I mastered that competency to the level I aimed for?”

(Assessor 14)

Other assessors measured students’ level of competence by their ability to reflect on their own competencies. Consequently, they started by reviewing the student’s self-reflections, considering it a no–no when a student was not aware of his or her competence in one or more areas:

Because I think it is really very good if a person has self-knowledge about his own weaknesses. I appreciate that a lot, because that is where it all starts. When you yourself want to improve. When other people feel that you have weaknesses in a given competency and you yourself disagree, I think that is – that is a scary person.

(Assessor 11)

A final distinction we found between assessors’ interpretations of ‘competence’ was reflected in the way they weighted and valued the various CanMEDS competency domains in the portfolio. While most assessors, regardless of portfolio version, specifically targeted ‘medical expert’, ‘manager’, and ‘communicator’ competencies and scanned the remaining competencies, others sought to bring into focus the full range of competencies. This latter tactic allowed them to differentiate between students, as collecting valuable feedback on the less well defined competencies (e.g., health advocate or scholar) is quite a challenge for students. Hence, students who were able to do so and reflected on this evidence were considered to be above average and eager to learn:

[…] in the domain of health advocate, for example, if someone already understands that there is more to being a health advocate than just, well, repeating to someone that he should quit smoking. Who also identifies where those problems - those difficult situations are in the workplace. Well, that’s where I see an above-average person […] Well, of all students, I think, the above-average ones know what to fill out under ‘scholar,’ ‘professional,’ and ‘health advocate,’ under those less obvious [competencies] that is.

(Assessor 7)

### Differences in how assessors construed the portfolio evidence

Throughout the entire judgment process assessors lent their own meanings to the evidence included in the portfolio, leading to different inferences about the student’s competence and attitude. More specifically, based on the same pieces of information assessors drew different conclusions, for instance about students’ responsibility for their competence and achievement: An insufficient rating on a workplace-based assessment of a specific student, was construed by one assessor as the result of a lack of knowledge, while another attributed it to insecurity. Conversely, sufficient ratings over a prolonged period of time were construed as underperformance by some, since they had learned from experience as an assessor and supervisor that written assessments were generally on the positive side be as supervisors eschew failing a student:

And then I realize how hard it is to deliver that emphatic ‘insufficient.’ You are inclined to soften the blow for that person and then it is very difficult to give a one^1^ or a two, so you give a three. And that is why I am cautious when someone only scores threes. Really, because those threes could also be taken to mean a score of one to three, instead of a three.

(Assessor 12)

Yet other assessors felt they could not make an accurate interpretation of the assessment feedback in the portfolio without having some background information about the student. These assessors were also interested in the student’s extracurricular activities, interests, and hobbies. In an effort to know more about the student’s background, some assessors actually commenced their portfolio review by reading the student’s curriculum vitae:

Anyway, the reason why I do that from time to time is that you look, what kind of side jobs did this person have? Is he a member of an association? Is it someone who looks around him? I’d rather have people who travel, play music and sports, look around them, speak foreign languages, read three newspapers, and pass by the narrowest margin. Than someone who receives outstanding grades but does not leave his room, you see? Then it is not about what I want, but it is about, I just want to have the full picture of such a person.

(Assessor 9)

The above-named different interpretations of performance data led to varying inferences about a student’s performance and to equally dissimilar judgments about specific components in the competency framework. Although we described each of the three inter-assessor differences separately, they actually acted in concert to mediate assessor’s decision-making process. When reading portfolio A, for instance, assessor 1 attached great importance to the ‘medical expert’ role and therefore specifically looked for all portfolio evidence about this competency. Consequently, this assessor believed that the most reliable information about this competence came from doctors and could be found in the workplace-based assessments. In the end, the assessor inferred that the student’s competence was problematic since the student lacked competence in the medical expert domain. Assessor 2, however, who read the same portfolio, was more interested in determining the student’s progress. This assessor believed that by reading the comments in the multisource feedback he would be able to do so, because this feedback contained opinions of multiple people about the student’s competence over a longer period of time. Although acknowledging that the student should pay attention to the medical expert competency, the assessor was not concerned because the student had improved considerably during the clinical rotation.

## Discussion

The present study has sought to enhance our understanding of how assessors form judgments of students’ professional competence based on the evidence collated in a competency-based portfolio.

Our findings suggest that assessors’ information processing is characterized by iterative phases of acquiring, organizing and integrating information. Previous research on rater cognition has found similar phases (Gauthier et al. 2016). Although all assessors had their unique approaches, as evidenced by differences in their credibility judgments, performance theories, and inferences, they eventually reached the same overall judgments. This finding is consistent with research by Gingerich et al. (2014) who investigated what proportion of variance in physicians’ mini-CEX ratings could be attributed to physicians’ development of one of a few distinct social impressions about the resident: While raters provided different causal explanations for their judgment, subgroups of raters were making similar judgments.

We also found that assessors’ selection of evidence and the extent to which they let this evidence influence their judgment were strongly governed by their beliefs about the credibility of the portfolio evidence. This judgment of credibility is not exclusive to assessors, as it resulted from a study by Watling et al. (2012) who asked students to reflect on experiences that had influenced their learning. When confronted with feedback, students judged the credibility of this feedback in order to decide which information they would use to inform their development. As with the assessors, the source of feedback played an important role in their credibility judgment. When students, for example, respected the individual for his or her clinical competence, they would more readily accept the feedback. Despite some research suggesting that people eschew providing non-anonymous feedback on a student’s underperformance for fear that it will affect their working relationship (Castanelli and Kitto 2011; Ingram et al. 2013), both research by Watling et al. (2012) and the present research demonstrate that it is vital to know the feedback source in order to be able to assess the information’s credibility.

Our findings also revealed that assessors held different performance theories which guided their beliefs about what they needed to know about a student to be able to make a well-informed decision. Confirming previous research where decision-making was based on direct observations (Ginsburg et al. 2010), the assessors in our study also based their judgments on aspects (such as student progress and self-reflections) that were external to the competency framework providing the portfolio structure.

Judgments based on direct observations inherently involve automatic decision-making processes. It has been demonstrated that automatic decision-making processes involving the categorization of people could lead to conversion errors and assessors’ inability to differentiate between competencies (Kolars et al. 2003; Macrae and Bodenhausen 2000). Furthermore, automatic decision-making involves the use of heuristic techniques used to speed up the process of finding a satisfactory, though possibly not optimal, solution. If similar decision-making problems have often been faced earlier, decision makers tend to use readily available strategies to arrive at a decision more easily (Tversky and Kahneman 1975). In our study, various automatic processes including use of heuristics seemed to play a role in assessors’ decision-making as well. Assessors, for example, automatically favoured particular feedback sources. Furthermore, their inferences were shaped by previous experiences: Assessors automatically assigned causal explanations to portfolio evidence based on earlier experiences with similar students. Although these automatic decision-making processes influenced the judgment process and caused differences in assessors’ reasoning behind their evaluations, assessors experienced no difficulty assessing the students’ competence. What’s more, their final overall assessments were in harmony, despite the differences caused by automatic decision-making processes.

In a previous study, Moonen-van Loon et al. (2013) tested the separate and composite reliability of three workplace-based assessment tools (mini-CEX, DOPS, and MSF) included in a resident portfolio. They demonstrated that, from a psychometric perspective, combining several workplace-based assessment tools in a portfolio can be a feasible and reliable method for high-stakes judgements. In addition to confirming these findings, our study suggests that, next to various types of workplace-based assessments and performance evaluations, including self-assessments and reflective writing in the portfolio adds information that is meaningful and important to assessors. Apparently, self-assessments and reflective writings provide assessors with information that cannot be inferred from workplace-based assessments. Another important contribution of our study is the observation that assessors feel need to contextualize the assessment by obtaining and interpreting more general information about the student’s background.

### Limitations

Several limitations are worthy of mention. First, we conducted this research at Maastricht University where a specific portfolio is used. Since portfolios differ substantially in content and format, it is advised that this study be replicated in other settings where different portfolio types are used.

Next, the think-aloud procedure inherently harbours a limitation in that various thought processes cannot be verbalized because they are either automatic or happen so quickly that there is no time to verbalize them (Charters 2003). Although the participants’ verbalizations seem coherent and complete, it should be taken into account that we might have not captured all thought processes. Furthermore, using think-aloud procedures has the risk of participants creating explanations to satisfy the researcher rather than reporting their actual thought processes. However, in addition to the think-aloud procedure, we also conducted semi-structured interviews in which participants were asked about the reasons why they made particular decisions. Also, theoretical saturation was reached after 12 participants indicating that important common aspects of participants’ decision-making are captured.

Third, the assessors in our study were not used to evaluating the student’s competence based on portfolio evidence alone: Usually, they also knew the student personally and had regular face-to-face meetings with the student. Although assessors did mention they missed this personal contact, they indicated to feel able to provide a judgment based solely on the portfolio evidence. This practice of providing judgments based exclusively on portfolio evidence, moreover, ties in with the idea that reviewers who take care of the summative portfolio assessment should not be the same as those providing the formative assessment. In this way the confidentiality of personal reflections is not compromised by the rigor and judgments necessary for making promotion decisions (Dannefer and Henson 2007). Findings from our research thus suggest that it is feasible to separate both roles.

### Practical implications

This study reiterates the importance of assessors explaining their judgments about students’ competence. Differences between assessors’ explanations suggest that decisions should not be made individually, but should result from group discussions. Although multiple assessors may reach the same general judgment about a student’s competence, they do differ in their judgments of individual competencies and the reasoning behind their overall judgments.

Also, discussing judgment policies of other assessors will make assessors aware of the fact that their method of assessment is not universally shared. It will help them to become acquainted with other views of competence and portfolio interpretation. This enables assessors to incorporate assessment practices of other assessors into their own assessment process, and to build ‘shared mental models’ for competence assessment.

Our findings suggest implications for assessor training. Assessor training should focus on raising assessors’ awareness of their own beliefs, performance theories, and inferences. If they gain more insight into their own decision-making process and get acquainted with those of other assessors their decision making may improve. Furthermore, training should focus on the effect of group member composition and group processes on the decision-making processes as described by Hauer et al. (2016).

Furthermore, since assessors have different approaches to the selection and use of portfolio evidence, it is important that portfolios be designed in such a way that they facilitate the selection of and navigation through the portfolio evidence. Captions are important as well, for they summarize the context in which the competency feedback was provided to the student (Van Tartwijk and Driessen 2009), helping assessors interpret the evidence and decide if and how they want to use it for their judgment.

## Conclusion

The present study described the process whereby assessors reach judgments, when reviewing the evidence collated in a competency-based portfolio. Assessors were able to form a judgment based on the portfolio evidence alone. Although they reached the same overall judgments, they differed in the way they processed the evidence and in the reasoning behind their judgments. Differences sprung from assessors’ divergent assessment beliefs, performance theories, and inferences acting in concert. These findings support the notion that portfolios should be judged by multiple assessors who should, moreover, thoroughly substantiate their judgments. Also, assessors should receive training that provides insight into factors influencing their own decision making process and group decisions. Finally, it was proposed that portfolios be designed in such a way that they facilitate the selection of and navigation through the portfolio evidence.

## Footnotes

1 On a mini-CEX form, performance in each competency area is scored on a scale from one (failure) to five (excellent).
